# The anterior esophageal region of *Schistosoma japonicum* is a secretory organ

**DOI:** 10.1186/s13071-014-0565-8

**Published:** 2014-12-10

**Authors:** Xiao Hong Li, Meg Stark, Gillian M Vance, Jian Ping Cao, R Alan Wilson

**Affiliations:** National Institute of Parasitic Diseases, Chinese Center for Disease Control and Prevention, 207 Rui Jin Er Road, Shanghai, 200025 China; Centre for Immunology and Infection, Department of Biology, University of York, Heslington, York, YO10 5DD UK; Cytometry Laboratory, Department of Biology, University of York, Heslington, York, YO10 5DD UK

**Keywords:** Esophagus, Vesicle, Transmission electron microscopy, Scanning electron microscopy, Antibody localisation, *Schistosoma japonicum*

## Abstract

**Background:**

The esophagus of blood-feeding schistosomes has been largely neglected although its posterior portion was designated as a gland decades ago. However, we recently showed it plays a pivotal role in blood processing. It is clearly demarcated into anterior and posterior compartments, both surrounded by a mass of cell bodies. Feeding movies revealed that erythrocytes accumulate in the anterior compartment before entering the posterior, indicating that a distinct process is executed there. We therefore investigated ultrastructural aspects and possible functions of the anterior region.

**Methods:**

The heads of adult *Schistosoma japonicum* were detached and prepared for both transmission and scanning electron microscopy to define the detailed ultrastructure of the anterior esophagus. Cryosections of heads were also prepared for immunocytochemistry and confocal microscopy to define the pattern of intrinsic host antibody binding in the anterior esophageal lining.

**Results:**

The anterior syncytial lining of the esophagus is highly extended by long, thin corrugations of cytoplasm projecting towards the lumen. Strikingly in the male worm, the tips of the corrugations are further expanded by numerous threads of cytoplasm, producing a spaghetti-like appearance in the central lumen. Flattened, pitted cytoplasmic plates are interspersed in the tangled mass of threads. Abundant, morphologically distinct light vesicles of varied size and contents are manufactured in the cell bodies, from where they traffic through cytoplasmic connections to the corrugations and out to the tips. Clusters of vesicles accumulate in expanded tips in males, together with occasional mitochondria whilst females have more mitochondria but fewer vesicles. The membranous contents of light vesicles are secreted mainly from the tips, but also from the sides of the corrugations. They coat the surfaces and then form organised self-adherent membrane figures when shed into the lumen. Host antibody binds strongly in a characteristic pattern to the anterior esophageal lining indicating that the secretions are highly immunogenic.

**Conclusions:**

We suggest that the anterior esophageal region is an independent secretory organ. The contents of light vesicles are released into the esophageal lumen via the tips of corrugation to interact with incoming blood. Our immediate task is to establish their composition and role in blood processing.

## Background

Adult schistosomes reside in the host blood stream, feeding exclusively on blood. The magnitude of this process in *S. mansoni* was revealed by *in vivo* tracer studies with Cr51-labelled erythrocytes [1]. A female worm ingested approximately 4.4× its body volume of plasma fluid per day, the male much less (0.2×) in spite of a larger size [2]. The blood is pumped by rapid grabbing contractions of the oral sucker, through the mouth, down a short esophagus to reach the blind ending gut caecum where terminal digestion and absorption take place. While much attention has focused on the enzymatic activities of the gut lumen and gastrodermis [3,4] the esophagus has been largely neglected. However, we recently demonstrated that it plays a pivotal role in feeding, with blood processing initiated there [5,6]. Erythrocytes are uncoated as they pass down the esophagus [5] while leucocytes become tethered in the posterior region to form a plug-like mass in which they are damaged or even destroyed [6]. Consistent with these functions, the esophagus has a complex, highly organised structure and is lined by syncytial cytoplasm, continuous with that of the tegument. The presence of a glandular structure around the posterior esophagus was established by electron microscopy more than 35 years ago [7-9]. It comprises a roughly spherical mass of cells linked to the lining syncytium by cytoplasmic connections through the muscle layer. The lining of the posterior region is expanded >25 fold in surface area by regular plate-like extensions of cytoplasm into the lumen [6].

Movies of *S. mansoni* feeding have revealed that blood ingestion is a multistep process [6]. Erythrocytes accumulate in an anterior compartment to form a bulge before passing as a bolus to a posterior compartment. This anterior compartment, like the posterior, is surrounded by a mass of cell bodies [6], although it has never been designated as a gland. One reason may be that in *S. mansoni* the syncytial cytoplasm of the anterior lining, whilst drawn out into longitudinal folds, nevertheless contained normal tegumental inclusions, namely discoid bodies and multilaminate vesicles [7-10]; see also the SEM images in [6]. There appear to be no equivalent studies of the *S. japonicum* esophagus and only two reports on the *S. japonicum* tegument [11,12]. From these it is apparent that the tegument of *S. japonicum* differs significantly in structure from that in the more exhaustively investigated *S. mansoni*. Additionally, while the posterior esophagus of *S. japonicum* appeared similar to that in *S. mansoni* [6], it was intriguing to observe a net-like appearance of the anterior esophageal lining in confocal images. This has prompted us to undertake a complete reappraisal of the cellular organisation and potential functions of the anterior esophageal region in *S. japonicum*.

## Methods

### Ethical statement

Animal care and all animal procedures were carried out in compliance with the Guidelines for the Care and Use of Laboratory Animals produced by the Shanghai Veterinary Research Institute. The study was approved by the Ethics Committee of the National Institute of Parasitic Diseases, Chinese Center for Disease Control and Prevention.

### Biological material

Cercariae of *S. japonicum* were shed from naturally infected *Oncomelania hupensis* snails collected from the wild in Anhui Province, China. Mice were experimentally infected percutaneously with 40 or 20 cercariae using the coverslip method [13]. Adult parasites were obtained by portal perfusion of the mice five weeks after exposure to 40 cercariae and 21 or 24 weeks after exposure to 20 cercariae, using RPMI-1640 medium buffered with 10 mM HEPES (Gibco, Life Technologies, Grand Island, NY, USA). These time points were chosen to represent newly mature adults and worms from chronically infected animals that had been subjected to prolonged exposure to immune responses. The worms were extensively washed in the same medium before tissue debris and any damaged individuals were removed under a dissecting microscope.

### Electron microscopy

The structure of the anterior esophagus was examined by transmission electron microscopy (TEM). Worms were fixed overnight at 4°C in 2.5% glutaraldehyde/4% paraformaldehyde in 100 mM phosphate buffer, pH7.4. Following two washes in phosphate buffer, they were post-fixed in osmium tetroxide for 2 hours, washed in H_2_O and dehydrated through a water-acetone series before single embedding in Spurr’s resin. Worm heads were then cut out of the resin and mounted in a transverse or longitudinal orientation on plastic stubs. Thick sections (0.5 μm) were cut and stained with toluidine blue for examination on a light microscope to identify the location of the anterior esophagus. Thin sections (70 nm) were then cut onto grids and post-stained with saturated uranyl acetate in 50% ethanol, and Reynolds’ lead citrate solution before examination in a Tecnai 12 BioTwin microscope (FEI, Hillsboro, OR, USA). Measurements of structures in micrographs were performed using the ‘Analyzing digital images’ package (Lawrence Hall of Science, UC Berkeley, CA, USA; http://www.globalsystemsscience.org/software/download) with the intrinsic scale as calibrator. Five male and five female worms were orientated for sectioning in TS at the two sampling times. The bulk of image series were obtained from one male and one female at week 5, and again at week 21. No age-dependent structural changes were observed in these worms.

The luminal surface architecture of the esophagus was examined by scanning electron microscopy (SEM). Worms were fixed in 4% formaldehyde in PBS at room temperature. The head region was then carefully sliced transversely into thin steaks (~ 30 μm) at ×35 magnification under a Leica EZ4 stereo microscope, using vannus scissors. The steaks were washed, dehydrated through an acetone series, critical point dried, individually positioned on stubs, and sputter-coated with 7 nm gold/palladium. They were examined using a JSM 6490-LV microscope for low magnification orientation and a JSM-7600 F for high-resolution images (both Jeol, Tokyo, Japan). More than 20 head steaks were examined by SEM; the majority of images come from four specimens with good transverse orientation.

### Confocal microscopy

Adult worms recovered at 24 weeks were fixed in 4% formaldehyde in PBS for four hours and then washed several times in PBS alone. The heads were then detached just posterior to the ventral sucker, as described for SEM, transferred to optimum cutting temperature (OCT) compound and orientated horizontally with a fine needle before freezing. Seven μm cryostat sections were cut and reacted with 1:100 dilution of AF488- labelled Goat-anti mouse IgG to detect intrinsic bound host antibody or with FITC-labelled Goat-anti hamster IgG (both Invitrogen) as a control for the specificity of staining. The musculature and nuclei were visualized by staining of f-actin with a 1:100 dilution of AF555-conjugated phalloidin (Invitrogen, Molecular Probes) plus a 1:400 dilution of 4′,6-diamidino-2-phenylindole (DAPI;1 μg/ml in PBS; Sigma-Aldrich, Poole, Dorset, UK), respectively, for 30 minutes. Optical slices were obtained using a LSM-710 confocal microscope (Zeiss, Cambridge, UK). Imaging conditions were as follows: DAPI: 405 nm diode laser with 405 main beam splitter (MBS); FITC/AF488: 3 mW argon laser with 488/561/633 MBS; Phalloidin AF555: 561 nm diode laser with 488/561 MBS.

## Results

### The tegument of S. japonicum has some unique structural features

**Figure 1:**
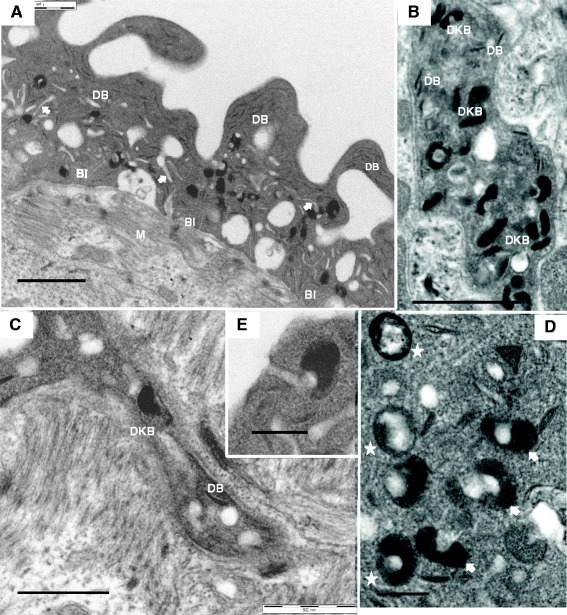
**TEM images of tegumental region of 5 week** ***S. japonicum*** **male. A**, Tegument in transverse section showing the homogeneous outer third layer with flaps of cytoplasm containing only discoid bodies (DB) and a central pitted region with a characteristic channel network (white arrows) as well as vesicles, whilst basal invaginations (BI) are evident in the innermost layer (M, muscle layer). **B**, Tegument cell body showing that two types of vesicle, dark bodies (DKBs) and discoid bodies, are manufactured here. **C**, Cytoplasmic connection between tegument cell body and tegument surface, with dark body and discoid body in transit. **D**, Dark bodies in tegument cell bodies reveal different profiles, e.g. top view (starred) and side view (arrowed). **E**, Dark body in tegument, docking via its opaque sphere to the end of a channel, so releasing its cargo. Scale bar: **A** 1 μm; **B** 1 μm; **C** 500 nm; **D** 200 nm; **E** 200 nm.

The syncytial tegument that covers the surface of adult male and female *S. japonicum* differs in a number of respects from that of *S. mansoni*. In the male, the outer one third is extended into flaps or lappets of relatively homogeneous cytoplasm. Below this lies a central region of pits, and finally an innermost layer into which numerous invaginations of the basal membrane extend (Figure 1A). Some of these basal invaginations are inflated and appear to encompass a small amount of debris. An unusual feature of the central region is a prominent network of short blind-ending membrane-bound channels running through the cytoplasm; these appear to connect to the sides or base of the pits (arrowed). In addition to mitochondria, two main types of inclusion are evident in the tegument cytoplasm: typical discoid bodies present throughout but especially prominent in the outer third; dark bodies, most abundant in the central region, some displaying a clear spherical zone (termed ring bodies by [11]). Examination of the tegument cell bodies lying beneath the muscle layer (Figure 1B) reveals that, in addition to rough endoplasmic reticulum and Golgi apparatus, the two main inclusions are abundant indicating their manufacture in this location. Further evidence is provided by the cytoplasmic connections running through the musculature from cell bodies to tegument syncytium, which also contain the two types of inclusion (Figure 1C). The dark bodies have an unusual structure, distinct from the multilaminate vesicles described for other schistosome species. They are bounded by a unit membrane with a flattened ovoid shape and dark, largely granular contents (Figure 1D). A lighter spherical mass of opaque material appears to be pressed down on one of the flattened faces of the ovoid, distorting the dark material to form a ‘nest’. This produces several distinct profiles in a thin section (Figure 1D). These dense bodies appear to dock with the end of a mid-region channel, via the opaque sphere (Figure 1E), presumably to release their contents into the channel from where these travel to the pits and tegument surface. The tegument of the female is much thinner than that of the male (< 2 μm) without the zonation but containing the same inclusions, plus the network of channels.

### The layout of the anterior esophagus reveals novel features

**Figure 2:**
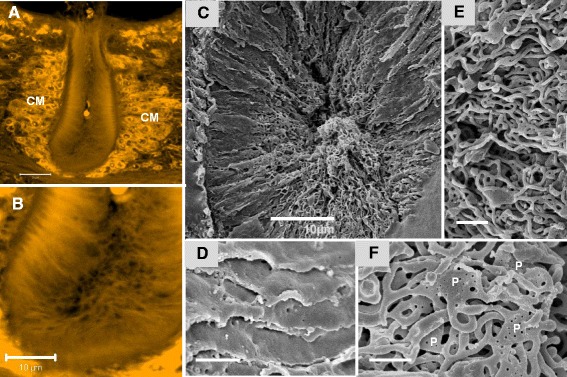
**Layout of the anterior esophageal region of** ***S. japonicum*** **adult male.** Confocal images stained with Langeron’s carmine: **A**, Illustrates the cell mass (CM) surrounding the anterior esophageal compartment. **B**, reveals the basal striated appearance of the lining and a central branched network of threads. SEM images: **C**, anterior esophageal lining showing its highly complex ultrastructure, including long, thin corrugations of cytoplasm extending from the base and central spaghetti-like cytoplasmic threads; **D**, corrugations are revealed as smooth thin layers of cytoplasm with a few tiny pits in the surface; **E**, tips of corrugations showing that the cytoplasmic threads branch frequently to form a net-like structure. **F**, Higher magnification showing cytoplasmic threads connecting flattened, pitted plates of cytoplasm (P). Scale bar: A 20 μm; B&C 10 μm; D 1 μm; E 2 μm; F 1 μm.

The overall layout of the anterior esophagus of *S. japonicum* conforms to that already described for *S. mansoni*, comprising a syncytial lining, an investing lattice of muscle fibres, and a ball of cell bodies (approximately 700 in the male, fewer in the female; Figure 2A). However, one principal difference concerns the lining syncytium, which is greatly increased in surface area by highly corrugated projections of cytoplasm, up to 20 μm long from base to tip in males (Figure 2B, and D) and proportionally shorter (only 5 μm) in females. SEM reveals that the corrugations, although reminiscent of the plates in the posterior esophagus, are very thin with smooth surfaces (Figure 2C). The most striking feature is that their tips are enormously extended by threads of cytoplasm, giving the appearance of a heap of spaghetti in the esophagus lumen (Figure 2D). Closer inspection shows that the cytoplasmic threads branch frequently and are anchored at their ends to the corrugations (or plates; see below), with only a few blind fingers (Figure 2E, and F). Interspersed at different levels within the tangled mass of threads are flattened plates of cytoplasm, which at high resolution are seen to be dotted with minute pits (30–40 nm diameter) spaced approximately 182 nm apart (Figure 2 F).

### The anterior esophageal cell bodies manufacture a novel inclusion

**Figure 3:**
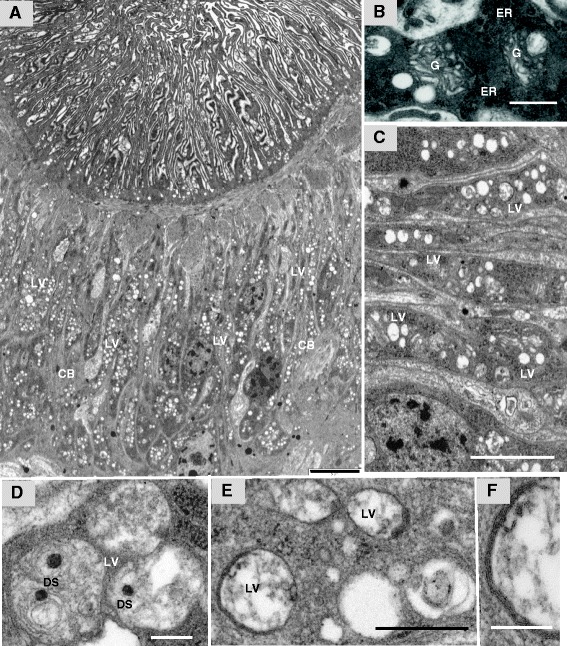
**TEM of anterior esophagus cell bodies. A**, Transverse section of anterior esophageal region, showing greatly extended lining cytoplasm while massive numbers of light vesicles (LV) are present in the elongated cell bodies (CB). **B**, Higher magnification of the cell bodies with abundant rough endoplasmic reticulum (ER) and Golgi apparatus (G), indicative of light vesicle manufacture. **C**, Anterior esophagus cell bodies showing light vesicles of variable size and appearance. **D**, Light vesicles with central dense spheroidal (DS) material. **E**, Light vesicles with material applied to the outer bounding membrane. **F**, High magnification of light vesicle reveals that this material is composed of membranous lamellae. Scale bar: A 5 μm; B 500 nm; C 2 μm; D 200 nm; E 500 nm; F 200 nm.

A second difference of the anterior esophagus, readily apparent from the electron micrographs, is that the cell bodies in *S. japonicum* do not appear identical to those beneath the tegument, and do not manufacture the same proportions of cytoplasmic inclusions for export to the esophageal lining (Figure 3A). These cell bodies are very elongate (up to 45 μm) tapering down to the point where their cytoplasmic connections run through the muscle lattice, and are 4.5 μm at their widest, to accommodate the nucleus (Figure 3A). Rough endoplasmic reticulum is abundant, and Golgi bodies are frequent, indicative of protein synthesis and packaging for export (Figure 3B). Indeed, nucleoli are also prominent in confocal images (Figure 2A) indicating active ribosomal biogenesis. The cytoplasm contains large numbers of vesicles (mean size 0.35 × 0.24 μm in males, and 0.42 × 0.39 μm in females) quite distinct in appearance from the dark bodies of the tegument, with sparse contents, hereafter termed ‘light vesicles’ (Figure 3C). Close inspection reveals that they are not all identical. Some are almost devoid of contents; others contain varying amounts of granular material (Figure 3D, and E). A proportion contains membranous lamellae, which may be either adherent to the bounding membranes (Figure 3D, and G) or present as loose whorls in the centre accompanied by a prominent single dense granule (63 nm diameter; Figure 3E).

### Light vesicles are transported to the leading edge of the luminal corrugations

**Figure 4:**
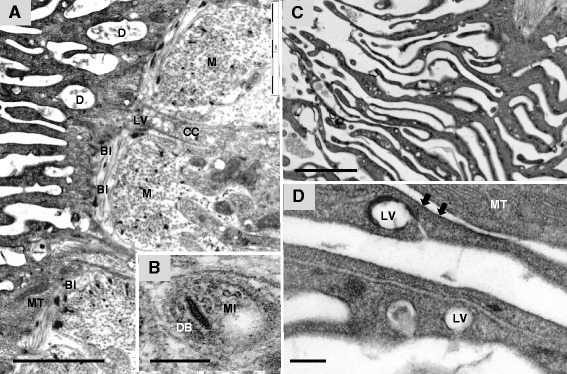
**Traffic of light vesicles. A**, Cytoplasmic connection (CC) between anterior esophagus cell bodies and basal corrugations, showing light vesicles (LV) in transit; debris (D) is evident between the corrugations presumably derived from released content of light vesicles (BI, basal invagination; M, muscle layer; MT, mitochondria). **B**, Transverse section of cytoplasmic connection reveals microtubules (MI) potentially involved in vesicle transportation (DB, discoid body). **C**, Anterior esophageal lining showing clusters of light vesicles distributed all the way down the tips of the corrugations. **D**, Light vesicles near the base of corrugations apparently emptying their cargo to the exterior; mitochondria are also evident whilst the surface is coated by membranous material (arrowed). Scale bar: A 5 μm; B 500 nm; C 2 μm; D 200 nm; E 500 nm; F 200 nm.

The cell bodies of the anterior esophagus are linked to the syncytial lining by narrow cytoplasmic connections through which light vesicles and occasional discoid bodies appear to be in transit (Figure 4A). Transverse sections through these connections reveal microtubules, which are probably part of the molecular-motor machinery for vesicle transport (Figure 4B).

The lining of the anterior esophagus comprises a thin basal layer of cytoplasm from which numerous corrugations extend; light vesicles and mitochondria are also present. This syncytial layer is not comparable in appearance to the tegument that lines the oral sucker and covers the worm body. In places, the lining corrugations are extremely narrow (50 to 70 nm), barely wide enough to accommodate two unit membranes and a little intervening cytoplasm (Figure 4C). In wider regions (180 to 500 nm, mean 275 nm) central parallel membranes 28 nm apart, denote the presence of basal invaginations (Figure 4D), similar to those described for the plates of the posterior esophagus; these invaginations run for 40 to 60% of the distance up each corrugation from its base. In the wider segments, clusters of light vesicles are apparent and reach all the way to the leading edge, implying they must be in transit (Figure 4C). Surprisingly the light vesicles within the corrugations appear smaller (mean size 0.17 × 0.14 μm in males, 0.18 × 0.13 μm in females) than those in cell bodies and the expanded tips. Mitochondria are also evident in the cytoplasm, together with sparse discoid bodies (Figure 4D). Both debris and membranous material are present between the corrugations (Figure 4A). Plausibly this material originates from the light vesicles, some of which can be seen fusing with the bounding membranes of the corrugations in this region to release their cargo (Figure 4A and D).

### Secretions are released from the expanded leading edges of the corrugations

**Figure 5:**
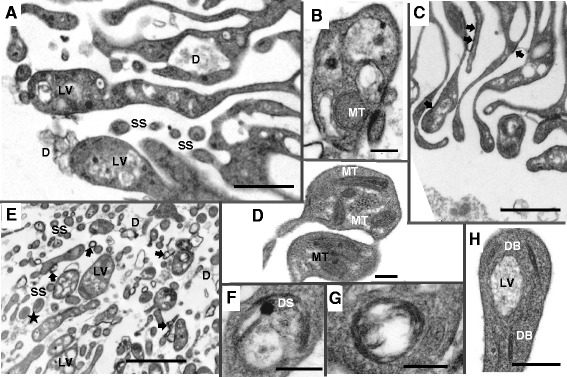
**Tips of anterior esophagus luminal corrugations.** A to B, E to G are from adult male; C ,D and H are from female; A and C are to scale. **A**, Clusters of light vesicles (LV) are evident inside expanded tips of adult males; debris (D) is frequently seen either attached to the surface of corrugations or present between them. Small spheroids (SS), representing sections of cytoplasmic threads, are devoid of vesicles. **B**, High magnification of the tip of a male corrugation showing four vesicles in different profiles, together with a single mitochondrion. **C**, Smaller expanded tips of female anterior corrugations, most of which contain only one light vesicle; membranous material (arrowed), evident between corrugations, is attached to the outer surface. **D**, High magnification of female corrugation tips, showing presence of one or two light vesicles but more prominent mitochondria (MT). **E**, A general view of male corrugation tips. Small spheroids represent transverse section of cytoplasmic threads lacking vesicles but containing the occasional mitochondrion (starred). Other tips are expanded to accommodate a cluster of vesicles; debris (D) is common in the lumen, much of it membranous (arrowed) and adherent to the surface or to other debris to form irregular shapes. **F**, Light vesicle with whorls of membrane and a dense spheroid (DS). **G**, Light vesicle with layers of membranous material attached to the inner surface of the bounding membrane. **H**, Tip of a corrugation with discoid bodies (DB) and a single light vesicle. Scale bar: A 1 μm; B 200 nm; C 1 μm; D 200 nm; E 2 μm; F 200 nm; G 200 nm; H 200 nm.

When viewed by TEM, the leading edge of many of the corrugations in male worms is sufficiently expanded to hold a cluster of vesicles of varying size (mean 0.4 × 0.29 μm) and appearance, spaced 0.4 μm apart (Figure 5A and B), plus occasional mitochondria. However, in females this expansion is less obvious with only one or two light vesicles present (mean size 0.45 × 0.33 μm), but a predominance of mitochondria (Figure 5C and D). The approximate surface area of the tip expansions is > 1 μm^2^ in males and 0.3 μm^2^ in females (Fig 5B, D). In contrast, the numerous narrow cytoplasmic threads appear circular in profile (diameter 170 nm; Fig 5E) but still contain occasional mitochondria. Light vesicles containing whorls of membrane and those with a dense central granule are both evident (Figure 5 F and G); discoid bodies also reach as far as the expanded tips (Figure 5H). Although direct evidence of an act of vesicle fusion is difficult to capture, there is a strong likelihood that the light vesicle contents are discharged from the leading edge to interact with constituents of incoming blood.

**Figure 6:**
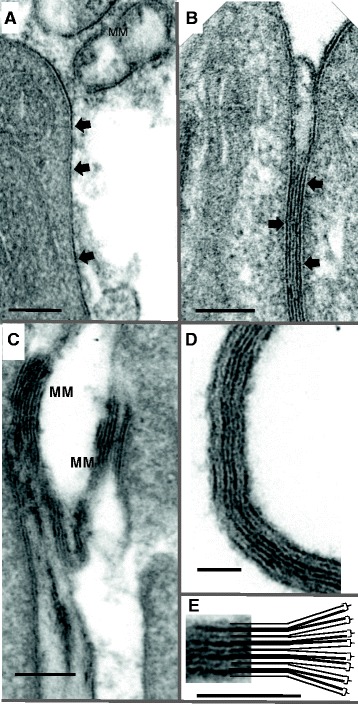
**Characteristics of the membranous coating on the corrugation surfaces. A**, Membranous material (MM & arrowed) covers the whole surface of the corrugations, and is also evident free in the lumen. **B**, Membranous material trapped between two adjacent corrugations, the alternating black and white stacks giving a tramline appearance. **C**, Layers of membranous material peeling off the surface of two adjacent corrugation surfaces to form the free aggregates in the lumen. **D**, High magnification of a free membranous stack showing the self-adherent properties of the constituents that produce myelin-like figures in the anterior lumen. **E**, A diagrammatic interpretation of the way in which the shed membranous layers combine to form an alternating pattern of black and white units. Scale bar: A 200 nm; B 200 nm; C 200 nm; D 200 nm; E 200 nm.

The whole surface of the corrugations from base to tip is covered with membrane-like material which can be seen both peeling off from the leading edge and free in the esophagus lumen (Figure 6A). To interpret the origins of this material, we use the model of the multilaminate tegument surface first proposed for *S. mansoni*, namely a plasma membrane overlain by a membrane-like secretion (termed a membranocalyx; reviewed in [14]). We infer that the membranous contents of the light vesicles are the source of the membrane-like material, released in quantity, which coats the surface of the corrugations and is then shed into the anterior esophageal lumen. This material has proved difficult to stabilise in all schistosome species using normal fixation and tissue processing procedures that rely on a progressive series of organic solvent extractions prior to resin embedding (Figure 6A). Fortuitously, it is more readily visualised when trapped between two adjacent corrugations (Figure 6B). Here the alternating black and white stacks are evident, and in some places layers are captured in the process of peeling off (Figure 6C). It is also apparent that the released material is self-adherent since it accumulates in even larger membranous stacks, several layers thick, in the esophagus lumen (Figure 6D). (This adherent property is apparent in the material when it is still within the parent vesicle; Figure 5 F). Careful inspection of the released membranous stacks at high magnification shows they are not of uniform electron density. Rather, there is a pattern of alternating broad and narrow dark lines (Figure 6D). This suggests an asymmetry in the individual units that comprise the membrane-like secretion. One leaflet of the secreted bilayer, probably the inner-facing one, is denser than the other that faces the external environment. When the membranous secretions peel off from the surface and associate, they apparently do so thick layer to thick and thin layer to thin, to create the observed alternating pattern of broad and narrow dark lines (Figure 6E).

### Host antibodies bind strongly to the anterior esophagus lining

**Figure 7:**
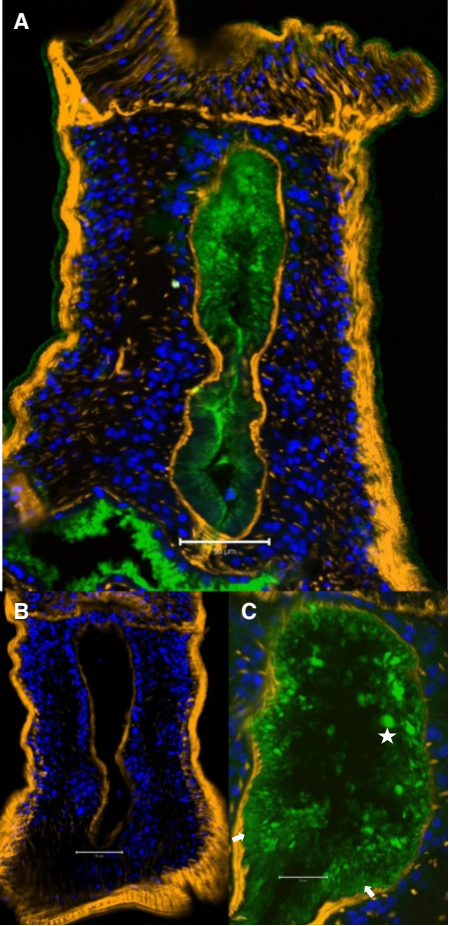
**Host antibodies bind to antigens in the esophageal lumen.** Cryosections were prepared from 24 week adult male worms from mice. **A**, Test, a longitudinal section showing localization of intrinsic mouse IgG (green), counterstained to highlight the musculature (orange) and nuclei (blue). Both anterior and posterior esophageal compartments are positive but with different patterns of reactivity; the staining of the anterior lining is focal and punctate whilst that of the posterior is mainly along the luminal edge of the plates. **B**, Control, reacted with AF488-labelled goat anti-hamster antibody; both anterior and posterior compartments are negative. **C**, High magnification image of anterior esophageal lining showing IgG distributed in intense focal blobs, numerous finer punctate spots and apparent striations (arrowed) that correspond to the individual corrugations. Most blobs are in the 2–3 μm range but one large sphere (6 μm, starred, host cell?) is an exception. Scale bar: A 50 μm; B 50 μm; C 20 μm.

Examination of longitudinal cryo-sections through male worm heads reveals a discrete pattern of IgG staining in the anterior and posterior esophageal compartments and transverse gut; a much weaker reaction is observed with the body surface tegument. The intensity of staining is stronger and more widely dispersed in the anterior compartment, apart from around the plug of leucocytes in the posterior lumen (Figure 7A). The lining plates of the posterior esophagus react only weakly except on the luminal edges. No positive staining was observed in control sections reacted with goat-anti hamster IgG (Figure 7B). Viewed at higher resolution, the staining pattern in the anterior esophagus is uneven with a few irregular patches of fluorescence up to 6 μm in diameter (Figure 7C). Behind these are numerous 0.5 to 1 μm fluorescent spots that give the whole lining a punctate appearance. Some of these patches of staining appear to be on the surface of the corrugations.

## Discussion

Our recent detailed study of the schistosome esophagus focused primarily on the structure and function of the posterior compartment surrounded by the esophageal gland [6]. Based on studies with *S. mansoni*, the prevailing view of the anterior compartment is that its lining and associated cell bodies are simply an extension of the body surface tegument. The syncytial cytoplasm has longer folds but nevertheless contains the normal discoid bodies and multilaminate vesicles present in the body surface [7,9,10,15]. The unstated assumption from these reports is that the anterior esophageal lining performs exactly the same functions as the body surface tegument, including evasion of the immune response. Our EM observations show that the anterior esophageal lining is not simply an extension of the tegument in *S. japonicum*. Furthermore, there are marked differences in ultrastructure of the surface tegument in the two species that have not been previously appreciated, particularly as the detailed description of the *S. japonicum* tegument by Sobhon & Upatham [11] has not attracted attention. Points of distinction in *S. japonicum* include: in male worms a clear division of the tegument cytoplasm into three zones; the presence of numerous ring bodies (our dark bodies); the occurrence of a system of membrane channels in the central zone. Similar but transient channels have been described in *S. mansoni* [16] as the sites where multilaminate vesicles fused with the plasma membrane to release their contents that formed the membranocalyx [17]. Our TEM images suggest that the channels likely play a similar role in *S. japonicum* but appear to be of a permanent nature. A more recent review [12] provided only low magnification images but noted the presence of “large membranous bodies of 150-200 nm diameter” in the tegument. It is reasonable to assume that these inclusions, irrespective of the terminology, represent the source of the membrane-like material that covers the tegument surface. In the anterior esophageal lining of *S. japonicum* the dark bodies are entirely replaced by the light vesicles whereas the discoid bodies are common to both surfaces.

While SEM has been used to characterise the architecture of the body surface in *S. mansoni* [15,18] and *S. japonicum* [11,12,19,20], and the gastrodermis in *S. mansoni* [5] our study is the first to use the technique for an ‘internal’ examination of *S. japonicum*. Strikingly, it revealed that the syncytial cytoplasm lining the anterior compartment was extended into thin corrugations up to 20 μm long in males, which terminated in threads of cytoplasm (cf. the plates of the posterior esophagus lining in *S. japonicum* which are up to 25 μm from base to tip). What might this remarkable cellular architecture achieve? Clearly the corrugations expand enormously the surface area of the anterior esophagus while the fringe of threads extends still further the potential interaction surface with ingested blood. Indeed it is possible, especially in the males, that the spaghetti-like threads intruding into the lumen are intended to entangle cells and retard their passage, allowing more time for interaction with esophageal secretions.

The mass of cell bodies surrounding the anterior esophagus are clearly a factory, primarily for the manufacture of light vesicles and to a lesser extent discoid bodies. If the relative proportions of the anterior and posterior cell masses are indicative of their physiological activity, then a one-third to two-thirds division of labour in the processing of ingested blood by the two compartments is likely. The heterogeneous appearance of the light vesicles themselves in the cell bodies, containing both membranous and granular material, implies a multiplicity of functions. Indeed, by analogy with the tegument it is likely they are the source of the membranous layer that coats the corrugations and is found free and aggregated in the anterior esophagus lumen. By inference from their distribution in the esophageal tissues, the light vesicles appear to traffic from their sites of manufacture at the Golgi apparatus of the cell bodies, via the cytoplasmic connections, along the lining corrugations to their tips, making these the main site for the release of vesicle contents. Given the thinness of the corrugations in many places, this traffic must occur along designated channels wide enough to accommodate the vesicles, accounting for the bunching of vesicles within the corrugations. The expanded corrugation tips of males containing clusters of vesicles are a major distinction from females with usually only a single vesicle. This could indicate that the male tips are a temporary storage site for vesicles, which accords with the recent suggestion that males feed intermittently but females continuously [2]. Mitochondria are more prominent in the tips of females than males, suggesting a greater need for energy at this location in the former. Indeed, if the voracity of the female *S. japonicum* is comparable to that of *S. mansoni* relative to their respective males, this could amount to at least a nine-fold greater ingestion and processing of blood by the female [1].

We were not successful in preparing the tiny female esophagus for SEM but high-resolution images of males revealed flattened, pitted regions, < 1 μm^2^ in surface area, amongst the spaghetti threads. It is tempting to equate these to the expanded tips of the corrugations seen in the TEM, with the inference that the pits could represent the potential sites of vesicle fusion with the plasma membrane. However, the spacing of the pits (182 nm) and the vesicles (400 nm) do not quite match up, even allowing for some shrinkage of tissues by critical point drying for SEM [21].

The presence of a central invagination within each corrugation is a feature shared with the more highly organised plates of the posterior esophagus. This arrangement is typical of transporting epithelia [6] and we have suggested that in the posterior esophagus, the plates might function to generate massive ion-fluxes into or out of the lumen. The same arguments can be applied to the anterior esophagus, although such a flux may not be linked directly to erythrocyte lysis since, in *S. mansoni* at least, that occurs in the posterior compartment [5]. Careful measurements showing that light vesicles within the corrugations were smaller in diameter than their counterparts in the cell bodies and corrugation tips, supports the idea of an ion flux. Assuming the vesicles are acting as osmometers, then the reduction in volume suggests that in transit to the tips they pass through a region of higher osmotic potential causing them to shrink. Such a gradient could be created by a flux of ions across the corrugations, generated by transporters situated in the membranes of the basal invaginations. It must be emphasised that the light vesicles contain granular material as well as whorls of membrane indicating that the contents may serve several functions. An obvious one is release of the membranous products to coat and protect the esophageal surfaces from immune attack, dealt with below. Another may be release of the granular component comprising proteins that interact with the incoming blood cells and plasma. Expanded tips of the esophageal lining, reminiscent of the structures we describe here, have been observed in the rodent blood fluke *Schistosomatium douthitti* [22]. Furthermore, the clusters of vesicles they contained were shown to be positive for acid phosphatase activity, suggesting they were lysosomes. We were unable to confirm acid phosphatase activity in the anterior esophagus in our study but this observation in a related species raises the possibility that *S. japonicum* is secreting lysosomal enzymes into the esophagus lumen. Certainly the morphology of the light vesicles is akin to that of primary lysosomes.

The similarity in anterior esophagus structure between *S. douthitti* and *S. japonicum*, the earliest divergent member of the genus *Schistosoma*, suggests that the production of light vesicles and secretion of their contents is a plesiomorphic (‘ancestral’) feature [23,24]. It is conceivable that in more derived lineages such as *S. mansoni* the anterior esophagus lining has converted to essentially normal tegument but whether this is so (and how it might relate to differences in the processing of blood between *S. mansoni* and *S. japonicum*) remains to be established. However, it should be noted that *S. japonicum* is a zoonotic fluke that can complete its natural life cycle in seven Orders of the Mammalia [25] whereas *S. mansoni* is essentially confined to a single species of primate.

Although the dark vesicles that supply the tegument surface with its membrane-like coating are replaced by the light vesicles in the anterior esophagus, the lining still displays the multilamellar characteristics of the tegument. From this we infer that in spite of originating from a morphologically different vesicle, this secreted membranocalyx functions as an inert protective barrier in the anterior esophagus, in the same way as it does over the tegument surface. The abundance of the membranous aggregates on the surface of the lining and in the lumen may indicate a fast turnover of this secreted layer, which could reflect the effect of antibody binding and sloughing. In this respect, the strong reactivity of the anterior esophagus with host IgG is surprising. It differed from our previous observations on *S. mansoni* worms from mice and hamsters where there was a uniform detection of host antibody, apparently confined to the esophagus lumen [6]. This suggests that cryosections are preferable to permeabilised whole worms for the generation of detailed high resolution images using the confocal microscope. Furthermore the body surface tegument of *S. japonicum* reacts only weakly for IgG implying that it is protected from antibody binding by its membranocalyx. However, if the worm has no option but to secrete proteins from specific sites on the tips of the esophageal corrugations, then such strong reactivity might be anticipated. Indeed, our high resolution images of worm head cryosections revealed that the pattern of intrinsic antibody binding on the anterior lining was punctuate, not continuously uniform. This indicates a marked heterogeneity of composition with only some points in the lining strongly recognized.

Our observations were made on worms from a murine permissive host where this bound antibody appears to cause no harm, perhaps precisely because there is rapid sloughing of targets. Nevertheless, it would be pertinent to discover the precise nature of the secretions emanating from the anterior esophageal vesicles since these are accessible to antibodies in a functional state before they can be subjected to proteolysis by the battery of hydrolases in the gut; this could make them good vaccine targets. Recently, a micro exon gene (MEG-12) has been identified as uniquely expressed in the anterior esophageal cell bodies of *S.mansoni*, the first such marker for these cells (Crusca et al., personal communication). Furthermore, its protein product has been implicated in the initial step of blood processing by interaction with incoming erythrocytes to destabilise the plasma membranes. Its novel site of expression in the anterior esophagus may indicate that the secretory inclusions of the lining syncytium in *S. mansoni* are not, after all, typical of the tegument. It would also be instructive to discover whether *S. japonicum* possesses a MEG-12 gene, and what other products are manufactured by the anterior esophageal cell bodies of both species.

## Conclusions

The schistosome esophagus is not simply a muscular tube that conveys blood from the oral cavity to the gut for digestion. In a previous study we showed that it is was divided into anterior and posterior compartments, with the ball of cell bodies that comprise the gland around the latter portion secreting proteins into the lumen to interact with incoming blood. We now show that, in *S. japonicum* at least, the smaller ball of cells around the anterior compartment also serves as a gland. The secretory vesicles manufactured in this region have distinct morphology from those of the posterior, suggesting a different composition. The cytoplasmic lining of the anterior compartment has a spaghetti-like appearance that may allow it to entangle incoming cells to increase their interaction time with the secretions. We suggest that the two masses of cell bodies should be designated the anterior and posterior esophageal glands in recognition of this distinction in morphology. We also show that the anterior compartment is the site for intense binding of host antibody, indicating that these secretions are highly immunogenic. Work is in progress to define the proteins manufactured in the anterior gland in order to understand better the role of the esophagus in blood feeding.
